# Does burst-suppression achieve seizure control in refractory status epilepticus?

**DOI:** 10.1186/s12883-018-1050-3

**Published:** 2018-04-21

**Authors:** Kanitpong Phabphal, Suparat Chisurajinda, Thapanee Somboon, Kanjana Unwongse, Alan Geater

**Affiliations:** 1 Neurology Unit, Department of Medicine, Faculty of Medicine, Prince of Songkla University, Hat Yai, Songkhla, 90110 Thailand; 2 Prasat Neurological Institute, Bangkok, 10400 Thailand; 3 Epidemiology Unit, Department of Epidemiology, Faculty of Medicine, Prince of Songkla University, Hat Yai, Songkhla, 90110 Thailand

**Keywords:** Burst suppression, Refractory status epilepticus, Midazolam, Outcome

## Abstract

**Background:**

The general principles in the administration of anesthetic drugs entail not only the suppression of seizure activity but also the achievement of electroencephalography burst suppression (BS). However, previous studies have reported conflicting results, possibly owing to the inclusion of various anesthetic agents, not all patients undergoing continuous electroencephalography (cEEG), and the inclusion of anoxic encephalopathy. This study aimed to analyze the effects of midazolam-induced BS on the occurrence outcomes in refractory status epilepticus patients.

**Methods:**

Based on a prospective database of patients who had been diagnosed with status epilepticus via cEEG, multivariate Poisson regression modules were used to estimate the effect of midazolam-induced BS on breakthrough seizure, withdrawal seizure, intra-hospital complications, functional outcome at 3 months, and mortality. Modules were based on a pre-compiled directed acyclic graph (DAG).

**Results:**

We included 51 non-anoxic encephalopathy, refractory status epilepticus patients. Burst suppression was achieved in 26 patients (51%); 25 patients (49%) had non-burst suppression on their cEEG. Breakthrough seizure was less often seen in the burst suppression group than in the non-burst suppression group. The incidence risk ratio [IRR] was 0.30 (95% confidence interval = 0.13–0.74). There was weak evidence of an association between BS and increased withdrawal seizure, but no association between BS and intra-hospital complications, mortality or functional outcomes was observed.

**Conclusion:**

This study provides evidence that BS is safe and associated with less breakthrough seizures. Additionally, it was not associated with an increased rate of intra-hospital complications or long-term outcomes.

## Background

Refractory status epilepticus (RSE) is defined as status epilepticus that cannot be controlled with an adequate dose of first-line and second-line antiepileptic drugs [1]. Refractory status epilepticus develops in approximately 30–40% of patients with status epilepticus [1].

This condition is associated with progressively increasing intrahospital mortality ranging between 19 and 67% depending on the study [1, 2]. Also, RSE patients experience functional impairment at discharge and long-term morbidity [1, 2]. The best management of refractory status epilepticus remains unclear. However, current guidelines recommend treatment with a continuous infusion of an anesthetic drug [1]. Available anesthetic drugs for continuous infusion include midazolam, propofol and pentobarbital. The major concerns of an anesthetic drug infusion are prolonged duration of mechanical ventilation, immobilization, hypotension, cardiac complications as well as propofol-infusion syndrome [2]. Midazolam has been shown to have a wide margin of safety and broad /therapeutic index, and be easy to use [1, 3].

A previous systematic review found that patients receiving pentobarbital had a lower frequency of breakthrough seizure as well as a lower rate of treatment failure [2]. However, these results were biased because of a lack of continuous electroencephalogram (cEEG) in the pentobarbital group and in those patients commonly experiencing subtle or non-convulsive seizures [2]. A recent guideline has recommended the initiation of an anesthetic infusion with cEEG to suppress seizure activity or burst suppression [1]. There are limited studies with conflicting results reporting on the effect of anesthetic drug-induced BS and its related clinical outcomes [1, 4–7]. However, different anesthetic agents have been used in the groups being evaluated. There is a scarcity of studies assessing the effects of EEG-BS in midazolam-treated RSE patients. The current study, therefore was conducted to examine the associations between midazolam-induced burst suppression and 1) the occurrence of breakthrough seizure, and 2) the occurrence of withdrawal seizure. Secondary outcomes were 1) intrahospital complications, 2) functional outcome at 3 months, and 3) mortality at 3 months.

## Methods

We compiled a systematic database of patients who had been diagnosed with status epilepticus by means of cEEG between from June 2005 and April 2016 at Songklanagarind Hospital, Thailand. Patients’ characteristics throughout treatment and follow up were collected from the clinical records by the first author. However, the treatment strategies were decided by attending neurologist and/or intensivist. Patients who continued to experience either clinical or electrographic seizure after receiving adequate dose of an initial benzodiazepine followed by some second acceptable antiepileptic drugs were considered to be RSE [1].

### Treatment protocol in our institution

The initial treatment of all status epilepticus patients consisted of intravenous diazepam (lorazepam is not available in our country) plus phenytoin (loading dose 20 mg/kg) or valproate (loading dose 20–30 mg/kg) or phenobarbital (loading dose 20 mg/kg) or levetiracetam (30–40 mg/kg). All patients treated with midazolam were on a mechanical ventilator (endotracheal tube insertion) and transferred to the intensive care unit. The midazolam dose was adjusted by the neurologist and/or intensivist base on the clinical observation of seizures and EEG monitoring. In our institution, midazolam is considered the first-line anesthetic drug for refractory status epilepticus as intensivists are familiar with its use in the management of agitated patients who are on a mechanical ventilator. Once the patient was seizure-free for 24 h, midazolam was tapered off over 6–24 h. The reduction rate was adjusted by the attending physician under cEEG monitoring to assess seizure recurrence. The causes of status epilepticus were categorized as central nervous system infection, metabolic disease, static brain lesion and antiepileptic drugs withdrawal. Patients were classified as having breakthrough seizure if any seizure occurred after 12 h of intravenous midazolam therapy or withdrawal seizure if any seizure occurred during the tapering off or within 12 h after the discontinuation of intravenous midazolam [8]. A good outcome was determined if, at 3 months, the patient’s condition returned to the clinical baseline or if the modified Rankin scale score was 0–2. A bad outcome was deemed if, at 3 months, the patient’s condition was evaluated to have a modified Rankin scale score of 3–6. Complications were categorized into: 1) respiratory complications (defined as the presence of hypoxemia, pulmonary edema, acute respiratory distress syndrome, and/or tracheostomy); 2) cardiac complications (defined as the presence of hypotension—mean arterial pressure < 70 mmHg or systemic blood pressure < 90 mmHg requiring a new administration of or an increase in the dose of vasopressor—new-onset arrhythmias, myocardial infarction or heart failure); 3) fever/infection (defined as the presence of a temperature of > 38.3°C or a positive culture needing antibiotics); 4) thromboembolic complications (deep vein thrombosis demonstrated by an ultrasound and/or pulmonary embolism demonstrated by lung CT-angiography); 5) gastrointestinal complications like ileus (defined as the absence of bowel movements in the absence of evidence of mechanical obstruction), intestinal ischemia (confirmed by surgical exploration), and gastrointestinal bleeding (both overt and covert demonstrated by an occult bleeding test), 6) hepatobiliary complications such as hepatitis (elevation of transaminases 3 times the upper limit of the normal values), pancreatitis (elevation of lipase > 3 times the upper limit of the normal values); and 7) acute kidney injury requiring renal replacement therapy. An electrographic seizure was diagnosed when paroxysmal EEG patterns with a discrete onset and evolution were present; periodic lateralized or generalized epileptiform discharges alone were not considered electrographic seizures.

The inclusion criteria were: 1) patients aged 15 years or older experiencing focal-onset seizures with a secondarily generalized seizure that cannot be controlled with an adequate dose of first-line and second-line antiepileptic drugs; 2) patients receiving midazolam for the treatment of RSE; and 3) patients undergoing cEEG. We excluded patients: 1) receiving an anesthetic drug other than midazolam and diagnosed with 2) epileptic syndrome; 3) psychogenic SE; 4) anoxic encephalopathy; or 5) complex partial SE.

The approval of our institution’s review board was obtained for the study.

### Statistical analysis

The basic patient characteristics and outcomes of BS and non-BS were compared using either the Pearson chi-squared or Fisher exact test. Prior to analysis, a Directed Acyclic Graph (DAG) was compiled to depict explicitly the potential relationships among predictors and outcomes. The associations between exposure and each outcome coded as yes/no were estimated using the Poisson regression adjusting for confounding variables (total effect) or confounding and intermediate variables (direct effect), as indicated by the DAG using the software DAGitty, version 2.3 [9].

The following variables were included in the DAG: EEG (BS), age, withdrawal seizure, breakthrough seizure, dose of midazolam, complications, etiology, status epilepticus severity score (SESS), time between SE diagnosis and start of midazolam, and outcome. The relationships between each of the variables were assigned by KP and AG based on knowledge regarding these associations from the literature review. Stata statistical software Version 14.1 (Stata Corp, College Station, TX) was used to analyze the data.

## Results

**Fig. 1:**
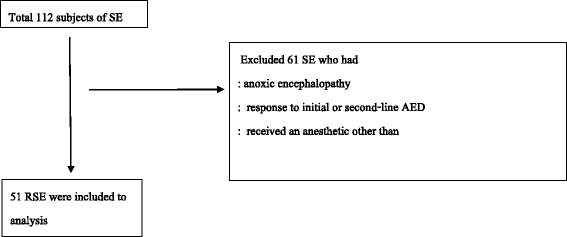
Flowchart of selection process

We recorded 112 patients with SE over the study period. Fifty-one non-anoxic encephalopathy RSE patients met the criteria and were included in the analysis. We excluded 61 patients who responded to initial antiepileptic drugs, had SE of an anoxic encephalopathy origin or received an anesthetic other than midazolam (Fig. 1). The median age was 49 years. Twenty-three patients (45.1%) were classified as having an acute encephalitis etiology. Eighteen patients (35.3%) had metabolic and 6 (11.7%) static lesions in the brain. There was a high variation in the time from the onset of SE to the treatment with midazolam ranging from 5 to 75 h (median 24 h). Ten patients had both breakthrough seizure and withdrawal seizure. Forty percent of patients had at least one breakthrough seizure and 33 % had at least on withdrawal seizure. Burst suppression was achieved in 26 (51%) patients; 25 (49%) patients had non-burst suppression on cEEG. The median maximum dose was 0.91 mg/kg. Seventy-two percent of all patients received a midazolam dose of ≥ 0.4 mg/kg. The median SESS was 4. In 84% of cases, seizure termination occurred within 60 min of initial midazolam infusion. Thirty-one (60.78%) patients had breakthrough seizure and 21 cases (41.18%) suffered withdrawal seizure. The mean hospital length of stay was 16 days (range 5 to 32 days). The average SESS was 4 (Table 1). Mortality among patients with RSE was 39.2%.

**Table 1:** Characteristics of patients in burst suppression and non-burst suppression groups

Variable	Burst suppression group	Non-burst suppression group	*P*-value^b^
	*n* (%)	*n* (%)	
Age (years)			0.136
< 30	5 (19.2)	8 (32.0)	
30–60	9 (34.6)	12 (48.0)	
> 60	12 (46.2)	5 (20.0)	
Sex			0.068
male	16 (61.5)	9 (36.0)	
female	10 (38.5)	16 (64.0)	
Etiology			0.353
encephalitis	13 (50)	10 (40.0)	
metabolic	10 (38.5)	8 (32.0)	
static lesion	1 (3.8)	5 (20.0)	
drug withdrawal	2 (7.7)	2 (8.0)	
History of epilepsy			0.067
yes	7 (26.9)	13 (52.0)	
no	19 (73.1)	12 (48.0)	
Number of antiepileptic drugs (before midazolam therapy)			0.35
2	10 (38.5)	5 (20.0)	
3	11 (42.3)	14 (56.0)	
4	5 (19.2)	6 (24.0)	
Status epilepticus severity score			0.27
3	7 (29.9)	12 (48.0)	
4	15 (57.7)	11 (44.0)	
> 4	4 (15.4)	2 (8.0)	
Dose of midazolam (mg/kg/day)			0.350
< 0.6	10 (38.5)	5 (20.0)	
0.6–1.2	11 (42.3)	14 (56.0)	
> 1.2	5 (19.2)	6 (24.0)	
Time to midazolam therapy (hours)			0.676
≤ 24	14 (53.8)	12 (48.0)	
> 24	12 (46.2)	13 (52.0)	
Length of hospital stay (days)			0.747
< 14	11 (42.3)	8 (32.0)	
14–21	9 (34.6)	10 (40.0)	
> 21	6 (23.1)	7 (28.0)	
Breakthrough seizure			< 0.001
no	19 (73.1)	1 (4.0)	
yes	7 (26.9)	24 (96.0)	
Withdrawal seizure			0.061
no	12 (46.2)	18 (72.0)	
yes	14 (53.8)	7 (28.0)	
Pulmonary complication			0.657
no	14 (53.8)	15 (60.0)	
yes	12 (46.2)	10 (40.0)	
Cardiac complication			0.789
no	20 (76.9)	20 (80.0)	
yes	6 (23.1)	5 (20.0)	
Fever/infection			0.301
no	14 (53.8%)	17 (68%)	
yes	12 (46.2%)	8 (32%)	
In-hospital mortality			0.029
alive	12 (46.2)	19 (76.0)	
dead	14 (53.8)	6 (24.0)	
Functional outcome at 3 months^a^			0.379
good	7 (58.3)	8 (42.1)	
poor	5 (41.7)	11 (57.9)	

^a^excluding dead patients

^b^Chi-square test

### Comparison between burst- vs non-burst suppression groups

The demographic and clinical characteristics of our participants are summarized in Table 1. There was no difference in baseline characteristics between the two groups. Regarding the disease etiologies of the patients who experienced successful burst suppression, they were: encephalitis (50%), metabolic (38.4%), static lesion (3.8%) and drug withdrawal (7.7%). In the non-burst suppression group, 48% of patients received midazolam within 24 h compared with 54% in the burst suppression group.

### Treatment outcome

The mean dose of midazolam in the burst suppression group was 0.9 mg/kg (range 0.21 to 2.5 mg/kg), and that of the non-burst suppression group was 1.23 mg/kg (range 0.3 mg/kg to 3.1 mg/kg); however, the difference was not statistically significant (*p* = 0.12). Breakthrough seizure was less often seen in the BS group than in the non-BS group. Its incidence risk ratio [IRR] was 0.30 (95% confidence interval 0.13–0.74). There was a tendency toward higher withdrawal seizure in the BS group as a direct effect of BS (IRR 2.04, 95% CI 0.76–5.47).

The incidence of in-hospital complications was comparable between the two groups. The common complications were pulmonary complications, cardiac complications and infection (43.1% vs. 21.6 vs. 39.2%, respectively). Interestingly, in the non-burst suppression group, hypotension and infection were less frequent than in the burst suppression group but without statistical significance. After adjusting for confounding factors, the achievement of BS was not associated with intra-hospital mortality, pulmonary complications, cardiac complications, fever/infection, thromboembolic complications, gastrointestinal complications, hepatobiliary complications or acute renal injury.

Concerning functional outcome at 3 months, according to the univariate analysis, a higher proportion of death in hospital occurred in the burst suppression than in the non-burst suppression group (*p* = 0.029, Table 1). However, this result was not confirmed after the adjustment for confounding factors (Table 2). The burst suppression or non-burst suppression was not statistically significant in terms of functional outcome (Table 2).

**Table 2:** Estimated adjusted incidence risk ratio (and 95% confidence interval) for the effect of burst suppression on various outcomes for which unbiased estimates could be made according to the conceptual causal diagram

Outcome	Effect	Controlled variable	Burst suppression	Incidence risk ratio^a^ (95% confidence interval)	*P*-value
Breakthrough seizure	Total = Direct	Etiology Status epilepticus severity score Dose of midazolam	Yes	0.30 (0.13–0.74)	0.009
Withdrawal seizure	Total	Etiology Status epilepticus severity score Dose of midazolam	Yes	2.04 (0.76–5.47)	0.157
	Direct	Breakthrough seizure Dose of midazolam	Yes	2.71 (0.89–8.25)	0.080
In-hospital mortality	Total	Etiology Status epilepticus severity score Dose of midazolam Time to midazolam therapy	Yes	2.02 (0.74–5.54)	0.170
	Direct	Breakthrough seizure Cardiac complication Dose of midazolam Etiology Pulmonary complication Fever/infection Status epilepticus severity score Time to midazolam therapy	Yes	3.25 (0.70–15.02)	0.131
Poor functional outcome at 3 months	Total	Etiology Dose of midazolam Time to midazolam therapy Status epilepticus severity score	Yes	0.75 (0.22–2.57)	0.646
Poor functional outcome at 3 months	Direct	Breakthrough seizure Withdrawal seizure Dose of midazolam Etiology Status epilepticus severity score Cardiac complication Pulmonary complication Fever/infection Time to midazolam therapy	Yes	0.13 (0.01–1.67)	0.116
Pulmonary complication	Total = Direct	Dose of midazolam Status epilepticus severity score	Yes	1.13 (0.46–2.82)	0.785
Cardiac complication	Total = Direct	Dose of midazolam Status epilepticus severity score	Yes	0.90 (0.25–3.27)	0.872
Fever/infection	Total = Direct	Status epilepticus severity score	Yes	1.38 (0.55–3.46)	0.498

^a^Adjusted for confounding and/or intermediate as required by the DAG

## Discussion

In this historical follow-up study of non-anoxic encephalopathy, refractory status epilepticus patients treated with midazolam, we found that breakthrough seizure was less often seen in the burst suppression group than the non-burst suppression group. The incidence risk ratio [IRR] was 0.30 (95% confidence interval 0.13–0.74). There was weak evidence of an association between BS and increased withdrawal seizure, but no association between BS and intra-hospital complications was observed. We also found a higher mortality in BS compared with non-BS patients (53.8% vs. 24.0%), but this difference was not confirmed to be statistically meaningful after controlling for confounding factors. Moreover, among surviving patients, no difference in functional outcome was seen at 3 months.

Burst suppression is defined as an electroencephalography finding consisting of a continuous alternation between of high-voltage slow waves (burst) and periods of depressed electrical activity [10]. Only sparse information is available with respect to the pathophysiological cellular mechanism of its pattern. It is, however, known that EEG bursts are associated with phasic synaptic depolarizing cellular potentials, occasionally crowned by action potential. A previous study has shown that suppression periods are due to the absence of synaptic activity among cortical neurons [11]. Medication, especially anesthetic agents titrated to attain burst suppression was associated with a significantly lower incidence of breakthrough seizure [4]. Nevertheless, whether EEG burst suppression is more effective than no electrographic seizure per se as an endpoint therapy for RSE remains unknown. Recently, Hernandez et al. conducted a retrospective study of 80 RSE patients in a neurological intensive care unit who were associated using video EEG. These authors also preferred general anesthesia and burst suppression was achieved in 78%. [12] The association between BS and seizure control in RSE has only been studied in small retrospective studies [2, 4]. In a retrospective study of pentobarbital-treated patients, the frequency of seizure relapse was 50% (6 in 12 patients) when BS was achieved compared with 15% (3 in 20 patients) in patients with a flat record (*p* = 0.049) [4] . However, this study suffered from differences in basic clinical characteristics of the population between the two groups, and a lack of continuous EEG monitoring performed throughout the barbiturate infusion as well as a multivariate analysis [4]. Claassen et al. conducted a systematic review of 28 articles (involving patients treated with different anesthetic drugs) and found that BS on EEG was associated with a lower frequency of breakthrough seizure, but it was not associated with the outcome. Interestingly, none of the patients who received midazolam achieved BS on cEEG in this review [2]. Our study was conducted to evaluate the effect of midazolam-induced BS. We found that BS on cEEG was associated with a lower frequency of breakthrough seizure; incidence risk ratio [IRR] 0.30 (95% CI 0.13–0.74, *p* < 0.01).

Overall, withdrawal seizure occurred in 21% of cases in our study compared with 40% of patients treated with midazolam [13–15], 20% of patients treated with pentobarbital [4, 16], and 10% of patients treated with propofol [17] reported in other studies. Fernandez et al. conducted a study to compare the effect of low dose versus high dose in patients with refractory status epilepticus and found that withdrawal seizure was less often seen in the high dose group [18]. Our study found that patients with BS were not statistically significant different compared with those of the non-BS group in this regard; IRR 2.04 (95% CI 0.76–5.47, *p* = 0.157) after controlling for confounding variables (total effect).

There were no differences between complications such as hypotension, cardiac complications and infection among the burst suppression and non-burst suppression patients [8]. In general, high doses of anesthetic drug infusion are associated with hypotension [18]. The use of pentobarbital to induce burst suppression has been associated with a higher incidence of hypotension compared with other anesthetics [4], but this finding was not supported by our study. We found no evidence that midazolam-induced BS was associated with pulmonary complications, cardiac complications, hepatobiliary complications, gastrointestinal complications, fever/infection, thromboembolism or acute renal injury.

Mortality in our study was 39% at discharge. At 3 months, 29.4% of patients had a good outcome. Other studies have found no association between therapy with intravenous anesthetic drugs and increased mortality. The systematic review by Claassen et al. reported a mortality rate of 46% in the midazolam group [4]. Previously reported mortality rates among RSE patients treated with low-dose midazolam and high-dose midazolam were 61 and 45%, respectively [4–6]. A recent retrospective study explored the association between different anesthetic agent-induced BS and their outcomes and found no association with poor outcome, including mortality, and poor functional outcome at discharge [6]. Our study was conducted to investigate the effect of midazolam on intra-hospital mortality, complication rate and functional outcome at 3 months. We found a higher mortality in BS compared with non-BS patients (53.8% vs. 24.0%, respectively), but this difference was not confirmed after controlling for confounding factors. Furthermore, among surviving patients, no difference in functional outcome was seen at 3 months.

The main limitations of our study were its small sample size, its being conducted in a single department, and its retrospective analysis. Few studies have investigated the association between BS and seizure outcome. They have included fewer than 35 RSE cases with cEEG [4, 6] and have not focused on a single anesthetic agent [5, 7]. Our investigation included more patients than previous studies, but it also has the limitation of being a retrospective study. However, a prospective study would be unethical due to the guidelines that recommend the endpoint of anesthetic induced-BS [1]. In fact, our data were collected prospectively and include many parameters. Due to the retrospective nature of our study, we did not control either the initial dose of midazolam or the time to the commencement of midazolam therapy after diagnosis. Finally, our study was conducted in a single department and did not focus on the characteristics of BS such as high epileptiform bursts. Future research should address these issues.

The strengths of our study consist in, firstly, its focus on a single anesthetic drug. Midazolam has been shown to have a wide margin of safety and a broad therapeutic index and is easy to use. Secondly, we based the statistical analysis on an explicit causal framework to ensure an appropriate co-variate adjustment in the regression analysis. The results of our study will be applied in the future management of RSE.

## Conclusion

In this historical follow-up study of the effect of the treatment with midazolam to induce BS in refractory status epilepticus using historical prospectively-collected data revealed that (1) burst suppression on cEEG was associated with a lower rate of breakthrough seizure but not with withdrawal seizure, mortality or functional outcome; and (2) burst suppression was not associated with hypotension, cardiac complications or infection.

## Abbreviations

BS: Burst suppression
cEEG: Continuous electroencephalography
DAG: Directed Acyclic Graph
EEG: Electroencephalography
EEG-BS: Electroencephalography- burst suppression
IRR: Incidence risk ratio
RSE: Refractory status epilepticus
SE: Status epilepticus
SESS: Status epilepticus severity score
